# A comparative study of pregnancy complications and outcomes for the years 1999 and 2004 at a rural hospital in South Africa: Implications for antenatal care

**DOI:** 10.4102/phcfm.v2i1.107

**Published:** 2010-10-18

**Authors:** Monjurul Hoque, Shahnaz Hoque

**Affiliations:** 1 Empangeni Hospital, Empangeni, South Africa; 2 Centre for Child and Adolescent Mental Health, University of Bergen, Norway

**Keywords:** Empangeni Hospital, pregnancy complications, pregnancy outcomes, pregnant women, rural KwaZulu-Natal

## Abstract

**Background:**

Detection and management of high-risk pregnancies, all the way through antenatal care, have been advocated as a high-quality mean of reducing maternal and perinatal morbidity and mortality.

**Objectives:**

This study reviewed the demographic variables, pregnancy and obstetric complications and perinatal outcomes for the years 1999 and 2004 in a rural hospital in KwaZulu-Natal Province, South Africa, with the aim of evaluating trends and gaps that may enhance appropriate strategies for improvement of antenatal care.

**Method:**

A retrospective comparative study, with representative samples of pregnant women, were randomly selected for the respective years 1999 and 2004. Descriptive statistics were calculated depending on measurement scale. A Z-test was carried out to assess the significant difference (*p* < 0.05) in proportions between pregnancy complications and outcomes of the groups. Multivariate logistic regression analysis was undertaken to determine the significant predictors for outcome variables.

**Results:**

The numbers of pregnancies among young women (< 25 years) increased significantly by 8% (*p* < 0.05) in the year 2004. Compared with 1999, the reduction in the numbers of pregnancies (1%) among higher parity (parity 5 or more) women in 2004 was remarkable. There were significant reductions of eclampsia, anaemia and post partum haemorrhage. Women with breech presentation were 3.75 times more likely to deliver preterm, and 5.45 times more likely to deliver low birth-weight babies. Similarly, women with pregnancy-induced hypertension were more likely to have preterm (OR = 3.50, 95% CI 2.83; 4.35) and low birth-weight babies (OR = 2.09, 95% CI 1.62; 2.71). Eclampsia was also a risk factor associated with preterm deliveries (OR = 6.14, 95% CI 3.74; 10.09) and low birth-weight babies (OR = 3.40, 95% CI 1.83; 6.28).

**Conclusion:**

This study suggests that further research is needed to find the causes of higher rate of teenage pregnancies and an increase in quality of antenatal care is more important in improving maternal and perinatal health. Training of staff to standard protocol and guidelines on antenatal care and care during delivery, and adherence to it, should be encouraged to improve maternal and child health in South Africa.

## INTRODUCTION

Ensuring better maternal and child health through reducing maternal and child mortality is the one of the UN Millennium Development Goals adopted by South Africa's (SA) health system.^1^ Detection and management of high-risk pregnancies, all the way through antenatal care, have been advocated as a high-quality means of reducing maternal and perinatal morbidity and mortality. Experience from developing countries has illustrated that making use of this practice has curbed pregnancy complications up to 60%.^2, 3^ Annually, approximately 530 000 women die worldwide from pregnancy related conditions and over 15% of pregnant women from developing countries experience pregnancy complications, resulting in negative maternal and perinatal outcomes.^4, 5^ Interventions in controlling the incidence of maternal and perinatal mortality and morbidity, considered effective in the context of developing countries, are not universally provided. A study conducted in Nigeria established that mothers who booked into health facilities for antenatal care and delivered in maternity units, experienced fewer complications and a lower maternal mortality rate than ‘unbooked’ mothers.^6^

Antenatal care includes, (1) education of pregnant women about pregnancy, labour, childbirth and newborn care, (2) prophylaxis for anaemia, malaria and immunisation against tetanus and (3) identification and management of pregnancy-related conditions such as anaemia, pregnancy-induced hypertension, diabetes and so on. Maternal health education through public health programmes should underline women's knowledge in very specific areas. These include:

- contextualised information about the appropriate age to become pregnant
- understanding ‘danger signs’ during pregnancy
- family planning
- appropriate ‘diet and exercise’
- the minimum gap between two pregnancies to ensure both baby and mother's sound health
- an awareness of HIV/AIDS to improve their chances of a good pregnancy outcome.

Despite all efforts to optimise the positive pregnancy and its outcome nationally and locally, maternal and perinatal mortality remains high in SA. For example, the anticipated maternal mortality ratio for SA was estimated as 147 per 100 000 live births, but it was actually higher (154) for the KwaZulu-Natal (KZN) Province in 2004.^7^ The perinatal mortality rates for KZN and SA were 26.5 and 37.5 per 1000 live births, respectively, in 2006.^8^

Maternal health programme efforts, particularly in the rural areas of the developing countries, are often poorly designed and quite deficient in reaching the target population.^9^ Previously, rights to utilise benefits of maternal health services was a major problem for the rural and Black communities of SA.^10^ Following the political transition in 1994, introduction of free maternal and child (< 6 years) healthcare (MCH) in the public health sector was very encouraging. It was reported from two rural areas of KZN (Hlabisa and Ubombo) that over 95% of pregnant women attend health facilities during pregnancy.^11, 12^ However, recent surveys of antenatal patients in Pretoria and in Greytown, KZN, have demonstrated that the majority of women only commence their antenatal care late in their pregnancy (i.e. in the second or third trimester).^13, 14^ Standard protocol and guidelines on antenatal care and care during delivery have been developed nationally and implemented throughout the country since 2002 to improve maternal and child health in South Africa.^15^

A few studies, particularly from rural KZN, have shown the demographic profile of pregnant women, along with pregnancy complications and outcomes. This study was undertaken with the objectives of measuring and comparing demographic profiles of pregnant women, specific pregnancy and obstetric complications and indicators for perinatal outcomes for the years 1999 and 2004. Data were collected from a rural hospital in order to measure trends and gaps so that appropriate measures for the improvement of antenatal care could be taken.

## METHOD

### Study setting

Empangeni Hospital is situated in the Uthungulu health district (one of eleven districts) of KZN and consists of mainly Black, rural communities (more than 450 000 people), who speak isiZulu. The hospital is a 256-bedded maternity and neonatal care hospital that provides obstetric, gynaecological and neonatal health services to its population, in addition to serving as a referral centre for 14 rural clinics. It covers approximately 95% of deliveries and 20% of antenatal attendees for public institutions of the district. Antenatal care as well as care during delivery, are performed in the hospital and throughout the district, according to the national protocol and guidelines.15 Antiretroviral treatment facilities were not available during the study periods, but a programme for HIV transmission from mother to child (PMTCT) started in April 2002 at all the health facilities in the district. There were three specialists (obstetricians) and 15 trained medical officers (full-time), who ran a comprehensive maternity unit (antenatal, delivery and post-natal care) on a rotational basis. One pediatrician and five medical officers ran the neonatal unit. Anaesthetic supports were received from Ngwelezane Hospital, situated 5 km away from Empangeni Hospital. The specialists and medical practitioners of Empangeni Hospital visited all affiliated clinics in the sub-district on a weekly basis to support and train staff and manage complicated antenatal cases. At Empangeni Hospital, pregnant women were booked for antenatal care and received referrals (according to national protocol) from peripheral clinics, for both antenatal care and deliveries. The primary health care (PHC) clinics in the sub-districts provided antenatal services, while pregnancy complications and confinement were referred to Empangeni Hospital. All nursing staff (midwives) working at antenatal clinics and maternity wards in Empangeni Hospital and PHC clinics received orientation, in addition to receiving in-service training, based on the standard national protocol and guidelines for management of women during pregnancies and deliveries.

### Study design, sample selection and data collection

A retrospective comparative study was conducted among the women who delivered at Empangeni Hospital in 1999 and 2004 (January–December). Random samples of 3875 and 3912 women from the total of 8238 and 9964 for 1999 and 2004, respectively, were selected using computer-generated numbers. Data were gathered over a two-month period from January to February 2005. Data were collected from the maternity ward's delivery register, which was the only source of official data (manual data). The delivery register contained women's demographics (names, ages, addresses) and information about antenatal care and pregnancy complications (e.g. anaemia, pregnancy-induced hypertension [PIH], diabetes, ante-partum haemorrhage, eclampsia, obstetric, labour and perinatal information). Standard definitions of the conditions were used to diagnose the above-mentioned conditions, as stipulated in the national guidelines.15

The facts about obstetrics and labour that were routinely recorded in the register included:

- presentation of foetus during labour
- plurality
- method of induction
- augmentation of labour
- time of delivery (recorded in hours and minutes)
- mode of delivery (normal vaginal, assisted vaginal delivery using vacuum, forceps or operative caesarean section during delivery)
- complications of delivery (e.g. perineal and/or cervical tear, post-partum haemorrhage [PPH] etc.)
- side-by-side perinatal information, including birth weight and birth outcome of babies (live birth, still birth, Apgar score in 1 min and 5 min).

Attending midwives recorded this information during admission and after delivery. All the midwives working at the labour ward were oriented and received in-service training on filling in the labour ward register, as well as for the compilation of weekly and monthly summaries for the presentation at weekly and monthly perinatal mortality meetings held in the hospital.

### Data analysis

Relevant data from selected women were compiled from the maternity register book and entered into a spreadsheet, using Microsoft Excel 2003. Thereafter, the data were imported to SPSS 11.5 for Windows, for analysis. Descriptive statistics were calculated (central tendency measures, measure of spreading, frequency tables) depending on measurement scale. A Z-test was carried out to generate notable differences (*p* < 0.05) in proportions between variables of the groups. Multivariate logistic regression analysis was undertaken to determine the significant predictors for outcome variables. The variables included in the study were: rates of anaemia, PIH, gestational diabetes, malpresentation, pre-eclampsia, PPH, operative and assisted deliveries.

Prior written permission was obtained from the hospital policy and ethics committee to make use of the maternity register to conduct the study. No identification of patients or staff was required to present the results.

## RESULTS

The mean ages of pregnant women across the two periods were similar (24.61 years), although remarkable differences in age groups and parity were observed (Table 1). The proportions of teenage mothers were considerably higher in 2004 (17%), compared to 1999 (14%). A notable increase of pregnancy (8%) among mothers below 25 years of age was also noted. Women in this age group constituted 50% of the total number of pregnancies in 1999 and this increased to 58% in 2004. There was a momentous reduction (*p* < 0.05) of pregnancies among higher parity (parity 5 or more) groups for the year 2004 (4.26%), compared to 2004 (5.35%).

**TABLE 1:** Comparison of demographic information of the sample population of Empangeni Hospital for the years 1999 and 2004

Variables	Year 1999 (*n* = 3875) I	Year 2004 (*n* = 3912) ii	*p*-value i / ii
Mean age	24.61 (s.d. = 6.459)	24.62 (s.d. = 6.153)	NS
Teenager (< 19 years)	14.26	17.37	*p* < 0.05
19–24 years	35.41	40.52	*p* < 0.05
25–29 years	20.72	21.24	NS
30–34 years	12.18	12.7	NS
35–39 years	6.01	6.37	NS
40+ years	2.5	1.87	NS
**Parity**			
Parity nil	45	44.81	NS
Parity 1–4	49.65	50.93	NS
Parity 5 +	5.35	4.26	*p* < 0.05

s.d., standard deviation; NS, not significant.

Comparison of pregnancy and obstetric complications are given in Table 2. The rates that increased in 2004 were post-term and vacuum deliveries, compared to those for 1999. Those that were significantly lower in 2004, were incidences of eclampsia, the prevalence of anaemia at 36 weeks (or later at the gestational age), induction of labour and PPH. No significant variations were observed regarding the rates of ante partum hemorrhage (APH), PIH, gestational diabetes, multiple pregnancy, preterm delivery, caesarean section delivery (including emergency procedures), augmentation of labour; third-degree perineum tear and retained placenta during the two comparative periods. Table 3 illustrates the perinatal outcomes between the two study periods. Perinatal outcomes were measured in terms of low birth-weight (< 2.5 kg, LBW) at delivery, live birth, still birth, fresh still births (FSBs), macerated still births (MSBs) rates and mean Apgar Scores in 1 min and 5 min. The different perinatal outcome indicators were similar for the two study years.

**TABLE 2:** Comparison of pregnancy and obstetric complications of the samples for the years 1999 and 2004

Variables	Rates		*p*-value for differences of rate

	Year 1999 (*n* = 3875)	Year 2004 (*n* = 3912)	
**Pregnancy complications**			
Ante-partum haemorrhage	0.8	0.6	NS
Pregnancy-induced hypertension	8.2	7.2	NS
Eclampsia	1.3	0.8	*p* < 0.05
Gestational diabetes	0.2	0.1	NS
Anaemia	7.5	5.8	*p* < 0.05
Multiple pregnancy	2.2	2	NS
Preterm delivery rate	11.69	12.53	NS
Term delivery	85.7	85.12	NS
Post-term delivery	1.47	2.17	*p* < 0.05
**Mode of delivery**			
Vaginal normal	73.2	71.2	NS
**Vaginal assisted**			
Vacuum	2.2	3.7	*p* < 0.05
Forceps	0.3	0.5	NS
Cesarean deliveries	25.3	24.6	NS
Emergency caesarean deliveries	18.2	16	NS
**Delivery complications**			
Labour induced	5.1	3.4	*p* < 0.05
Labour augmented	1.7	1.9	NS
Third-degree perineal tear	0.3	0.4	NS
Post-partum haemorrhage	1.5	0.9	*p* < 0.05
Retained placenta	0.3	0.2	NS

NS, not significant.

**TABLE 3:** Comparison of perinatal outcomes of the sample women from Empangeni Hospital who delivered during 1999 and 2004

Variables	Year 1999 (*n* = 3875) i	Year 2004 (*n* = 3912) ii	*p*-value i / ii
Low birth-weight delivery rates (< 2500 gm)	13.2	14.4	NS
**Birth outcomes**			
Live birth	96.7	96.8	NS
Still birth	3.3	3.1	NS
Fresh still birth	1.4	1.1	NS
Macerated still birth	1.9	2	NS
**Mean Apgar score**			
In 1 min	7.98	8.07	NS
In 5 min	9.4	9.47	NS

NS, not significant.

Multiple logistic regression outputs are shown in the Table 4. In this analysis preterm delivery, LBW babies and FSBs were considered as dependent variables. Independent variables found to be significantly associated with the dependent variables, using the chi-square test, were: age of mothers, breech presentation of foetus, induction and augmentation of labour, PIH, eclampsia, and caesarean, vacuum and forceps deliveries. Breach presentation was a key predictor for preterm and LBW deliveries; a breech presentation is 3.75 times more likely to result in a preterm delivery and 5.45 times more likely to result in the delivery of a LBW baby. A probable reason for such observations could be related to the immaturity of the foetus. Mothers with previous caesarean deliveries were found to be twice as likely (OR = 2.26) to deliver FSBs. Induction of labour was instituted more frequently for preterm pregnancies (OR = 2.46) and thus resulted in more LBW babies (OR = 2.24) and FSBs (OR = 4.38). Pregnant mothers with PIH were more likely to experience preterm delivery (OR = 3.50) and LBW babies or deliveries (OR = 2.09). Eclampsia during pregnancy was a major risk factor for preterm (OR = 6.14) and LBW (OR = 3.40) deliveries, while forceps deliveries were more likely (OR = 5.79) to deliver FSB babies.

**TABLE 4:** Logistic regression output for outcome variables

Independent variables	Dependent variables								

	Preterm delivery			Low birth-weight			Fresh still birth		
		
	B	Sig.	OR (95% CI for OR)	B	Sig.	OR (95% CI for OR)	B	Sig.	OR (95% CI for OR)
Age	-0.03	0.00	0.96 (0.95; 0.98)	-0.01	0.16	0.98 (0.97; 1.01)	0.03	0.01	1.03 (1.01; 1.07)
Breach presentation	1.30	0.00	3.75 (2.59; 5.44)	1.69	0.00	5.45 (3.51; 8.48)	0.50	0.12	1.65 (0.86; 3.15)
Induction of labour	0.90	0.00	2.46 (1.82; 3.33)	0.80	0.00	2.24 (1.56; 3.21)	1.47	0.00	4.38 (2.75; 6.97)
Augmentation of labour	0.18	0.48	1.20 (0.72; 2.00)	0.16	0.56	1.17 (.67; 2.05)	1.47	0.00	4.34 (2.18; 8.66)
Pregnancy-induced hypertension	1.20	0.00	3.50 (2.83; 4.35)	0.74	0.00	2.09 (1.62; 2.71)	0.40	0.05	1.50 (0.99; 2.26)
Eclampsia	1.80	0.00	6.14 (3.74; 10.09)	1.22	0.00	3.40 (1.83; 6.28)	0.21	0.67	1.23 (0.45; 3.39)
Caesarean delivery	0.02	0.80	1.02 (0.85; 1.23)	0.30	0.00	1.36 (1.10; 1.67)	-1.31	0.00	0.26 (0.17; 0.42)
Vacuum	-0.92	0.00	0.39 (0.22; 0.71)	-1.05	0.00	0.34 (0.16; 0.72)	0.20	0.65	1.22 (0.50; 2.96)
Forceps delivery	-0.68	0.30	0.50 (0.13; 1.86)	-0.60	0.43	0.54 (0.12; 2.46)	1.75	0.01	5.79 (1.48; 22.64)
Constant	-0.71	0.07	0.49	2.61	0.00	13.62	0.52	0.49	1.68

B, regression coefficent; Sig., significant; OR, odds ratio; CI, confidence interval.

## DISCUSSION

This study was confined to pregnant women who delivered at Empangeni Hospital in 1999 and 2004. The idea of conducting such a study presented itself to the authors in 1999 and a period of 5 years was considered appropriate in terms of gathering adequate data to serve the purpose of comparison, therefore 2004 became the year for this research setting. Nonetheless, these data reflected large delivery information of the population of Uthungulu district, because the majority of the deliveries under public health facilities were conducted at Empangeni Hospital. However, during the two study periods, mothers who delivered at home or at private health facilities were not included in this study. Although a vast majority (more than 95%) of pregnant women was known to attend public health facilities for antenatal care in rural areas of KZN, the exclusion of deliveries at home and in private hospitals are considered as a limitation of the present study.^11, 12^ The proportion of pregnant women who delivered at public health facilities was not known, because maternity care is free of charge in public health facilities and, as such, encourages women to utilise these facilities for deliveries, thus one could expect a higher rate of utilisation by the target population. The retrospective review of records limited the availability of some study variables and consequently led to information bias. For instance, recordkeeping on HIV infection and maternal deaths were not registered properly; thus maternal mortality estimates were not done. In addition, the rate of HIV infection and its effects on perinatal outcomes were not estimated; incomplete and incorrect recording of observations and histories by the attending midwives may also have led to information bias. However, the midwives were trained and oriented with the labour ward register. Thus, it was assumed that such bias would be slight.

It is observed that there has been a significant increase of teenage pregnancies in Empangeni across the two study years (1999 and 2004) and this finding is consistent with the provincial rate for KZN.^8^ The rate of pregnancies among younger mothers (ages < 25 years) has shown a drastic increase of 50% in 1999 to 58% in 2004. The factors which might have contributed to this increase include the following: firstly, there has been a change in sexual behaviour in society, including early initiation in sex, secondly, non-use or poor use of family planning methods by the younger women and, thirdly, a possible tendency to become pregnant in order to obtain social benefits in the form of government-sponsored child support.^16, 17^ The higher numbers of pregnancies among younger girls may be a result of one of the above-mentioned factors, or a combination of two or more of these factors. In an antenatal care audit, conducted at the same hospital during 2004, it was found that 90% of those pregnant women had no income.^18^ Therefore, effective strategies are urgently needed to improve the socio-economic conditions of rural people, particularly pregnant girls who are still in school. Our findings regarding the demographic characteristics of our study subjects were similar to those of another study on the same (pregnant) population, in terms of age and parity.^19^

A significant reduction, 5.35% in 1999 to 4.26% in 2004, of higher parity (≤ 5) women, could have been due to successful adoption or implementation of effective family planning methods by the higher parity and older women and/or rapidly growing urbanisation in South Africa.^20, 21^ It is believed that urban dwellers, including women, have better access to health facilities, health education and contraception. It is thus assumed that city dwellers aspire to a higher standard of living. It is also assumed that urbanites are generally receptive to, and value, the idea of planning their careers, as well as their families. Women who pursue a higher education tend to delay motherhood until they are older and more established in their careers. As a result, an implication of this trend is that they have smaller families.

A substantial decrease in the prevalence of eclampsia (0.9%), anaemia (5.8%) and PPH, were observed during 2004, although the pattern of changes in pregnancy, obstetrics and perinatal outcomes were very slight. In the developing countries, the prevalence rates of eclampsia are known to vary widely, from 0.5% to 8%.^22^ It is a common risk because of the following reasons: poverty, a limited education that results in low literacy, lack of knowledge and awareness regarding health issues, and superstitious beliefs and practices, all of which prevent women from seeking medical care and advice during their pregnancy.^21^ These factors may prevail among the rural population to a larger degree in comparison to city dwellers.

Nevertheless, a recent study has shown a very encouraging finding based on an antenatal care audit that confirmed that almost all pregnant women attend antenatal care.^18^ The finding also revealed that the majority (88%) received antenatal care from PHC facilities, with an average of six antenatal visits before delivery. More than 95% of the women had routine clinical and laboratory examinations on every visit, as recommended by the national guidelines.^18^ Thus, it is assumed that the optimal management of essential hypertension and PIH were the key factors in probable prevention of eclampsia through effective antenatal care. It is likely that the provision of antenatal care by following the standard protocol and guidelines might have contributed to a reduction in the rate of eclampsia. Similarly, lower (though not significant) rates of APH (8%) and PIH (7.2%) were observed in 2004, compared to the findings of 1999 (APH of 6.2% and PIH of 8.8%). To reduce anaemia in pregnancy, prophylaxis with haematinics was given to all pregnant women, who were intermittently screened for the presence of anaemia (e.g. at the booking visit, thereafter at 28 weeks and again at 36 weeks). In addition, appropriate intervention were undertaken during the antenatal period, as required by the national protocol.^15^ There was no significant difference as far as multiple pregnancies were concerned, because these were related to biological factors specific to the women and, therefore, no changes were expected. The overall caesarean section delivery rates seemed higher for both periods and corresponded to the provincial rate of KZN.^8^

### Perinatal outcome

The perinatal outcomes during the two study years (1999 and 2004) remained the same, although an increased mortality rate was anticipated due to the high prevalence of HIV infection among pregnant mothers in KZN.^23^ Efforts such as developing and implementing policies and standard guidelines to manage medical conditions, free access to public health facilities, local strategies (training health care workers), and ensuring better provisions of care (establishing and upgrading clinics and health facilities by the national, provincial and local health authorities) have had a positive impact on perinatal mortality. A formal evaluation of these efforts is necessary for such inference and extremely important for public health initiatives.

Preterm delivery rates were similar during the comparison periods, but at a higher rate (> 11%), compared to developed countries during the same period.^24^ Preterm delivery is the most important cause of perinatal mortality in the developed world.^25^ The prevalence of preterm delivery in first-world countries is between 6% and 10%.^24^ No obvious causes of preterm delivery have yet been established, but several etiological risk factors have been identified as non-obstetrical causes for preterm birth. These include poor socio-economic status, maternal malnutrition and/or smoking, illiteracy, maternal age below 20 years and over 35 years, smoking, and trauma are.^26, 27^ The obstetric risk factors found to be associated with preterm deliveries are: cervical incompetence, multiple pregnancies, short birth intervals, pre-labour premature rupture of membrane and previous preterm deliveries.^27, 28^ A number of other medical conditions have also been associated with preterm birth and these include diabetes mellitus, urinary and genital tract infections, HIV infection, and psychological stress.^29^ Some of these factors were prevalent in our present study population, especially in cases where poor socio-economic conditions and higher rates of HIV infection were present.^23^

Low birth-weight delivery rates, though, remained at the same high level for both years (> 13%). However, this rate was lower when compared to many other African countries (e.g. 18% in Tanzania in 1999 and 19% in Kenya in 2004), but higher than in, for instance, Brazil in 1996 (9%), which has a comparable economy to SA.^30–32^ Well-known factors for LBW delivery are premature delivery and intrauterine growth retardation. Personal (genetic, prior premature birth, age), social, environmental and medical risk factors (e.g. shorter pregnancy intervals, HIV infection, poor maternal nutrition, smoking), have been found to be associated with low birth weight in different parts of the world.^25^ These factors prevail in South Africa, particularly in rural KZN. In addition, an increasing rate of HIV infection among pregnant women in KZN (20% in 1996 and 37.5% in 2004) has been observed in this study.^23^

In South Africa, after the political transition in 1994, access to healthcare services, particularly maternal and child healthcare, became free of charge at the public health facilities in order to remove financial barrier to health services and thereby ensuring better access. As such, one can assume that pregnant mothers attended and received the standard of care, without any prohibiting factors preventing them from seeking the care and support they may need. Higher attendances and improved standard of care thus may have improved overall pregnancy outcomes in the present study population.

## CONCLUSION

The prevalence of pregnancy in teenagers and young women (< 25 years) has been found to be on the increase, but the rate is reduced among higher parity and older groups of women. Most of the pregnancy complications were found to have decreased over time (from 1999 to 2004) whereas the perinatal outcomes remained at the same level. Improvement of maternal and child health initiatives at different levels of public service delivery may have contributed to the effective management of pregnancy complications. Further research is needed to discover simple, yet effective strategies to combat negative perinatal outcomes.
